# Inflammation and viral infection as disease modifiers in schizophrenia

**DOI:** 10.3389/fpsyt.2023.1231750

**Published:** 2023-10-02

**Authors:** Hans C. Klein, Paul C. Guest, Henrik Dobrowolny, Johann Steiner

**Affiliations:** ^1^Department of Nuclear Medicine and Molecular Imaging, University of Groningen, University Medical Center Groningen, Groningen, Netherlands; ^2^Research and Education Department Addiction Care Northern Netherlands, Groningen, Netherlands; ^3^Department of Psychiatry, Otto-von-Guericke-University Magdeburg, Magdeburg, Germany; ^4^Laboratory of Translational Psychiatry, Otto-von-Guericke-University Magdeburg, Magdeburg, Germany; ^5^Laboratory of Neuroproteomics, Department of Biochemistry and Tissue Biology, Institute of Biology, University of Campinas (UNICAMP), Campinas, Brazil; ^6^Center for Health and Medical Prevention (CHaMP), Magdeburg, Germany; ^7^German Center for Mental Health (DZPG), Center for Intervention and Research on Adaptive and Maladaptive Brain Circuits Underlying Mental Health (C-I-R-C), Halle-Jena-Magdeburg, Germany; ^8^Center for Behavioral Brain Sciences (CBBS), Magdeburg, Germany

**Keywords:** schizophrenia, inflammation, viral infection, HSV-1, biomarker, drug target, personalized medicine

## Abstract

Numerous studies have now implicated a role for inflammation in schizophrenia. However, many aspects surrounding this aspect of the disease are still controversial. This controversy has been driven by conflicting evidence on the role of both pro-and anti-inflammatory factors and by often contentious findings concerning cytokine and immune cell profiles in the central nervous system and periphery. Current evidence supports the point that interleukin-6 is elevated in CSF, but does not support activation of microglia, resident macrophage-like cells in the brain. Furthermore, the mechanisms involving transit of the peripheral immune system factors across the blood brain barrier to central parenchyma have still not been completely elucidated. This process appears to involve perivascular macrophages and accompanying dendritic cells retained in the parenchyma by the chemokine and cytokine composition of the surrounding milieu. In addition, a number of studies have shown that this can be modulated by infection with viruses such as herpes simplex virus type I which may disrupt antigen presentation in the perivascular space, with long-lasting consequences. In this review article, we discuss the role of inflammation and viral infection as potential disease modifiers in schizophrenia. The primary viral hit may occur in the fetus *in utero*, transforming the immune response regulatory T-cells or the virus may secondarily remain latent in immune cells or neurons and modify further immune responses in the developing individual. It is hoped that unraveling this pathway further and solidifying our understanding of the pathophysiological mechanisms involved will pave the way for future studies aimed at identification and implementation of new biomarkers and drug targets. This may facilitate the development of more effective personalized therapies for individuals suffering with schizophrenia.

## 1. Introduction

A significant body of research points to a role of inflammation in schizophrenia (1, 2). The what, where and when of the exact hit the patient personally experiences remains obscure, although in a personal history this hit may occur anytime and many times, from embryogenesis to advanced age (3). This manuscript summarizes and tries to integrate the seemingly contradictory knowledge of hyper-inflammation versus hypo-inflammation occurring in patients already diagnosed with schizophrenia (4–6). We will follow-up on a current hypothesis regarding inflammation findings in the brain of schizophrenia patients: Downregulation of microglia activation in the brain and increase of IL-6 in CSF may be related to a failure of the functioning of peripheral regulatory T (Treg)-cells that operate at the blood brain barrier (BBB). Moreover, the state of inflammation found in a patient depends on the disease stage, whether it is before the first psychosis, during the first psychosis, during a next psychosis or a residual state of the psychiatric symptoms, usually manifested with negative symptoms and/or cognitive decline (7). However, different environmental triggers that may incite the inflammatory cascade may also influence the outcome. Current inflammatory hypotheses about schizophrenia revolve around microglia functioning in a more or less inflammatory or anti-inflammatory state, and accumulation of macrophages in the subependymal space (8), midbrain (9) and frontal cortex (10). In addition, they concern the potential of inflammatory impairment of adult neurogenesis (8, 11) and environmental gene interacting factors resulting in neuroinflammation (12).

Many aspects surrounding the role of inflammation in schizophrenia are still the subject of controversy. As examples, there has been conflicting evidence regarding a role of both pro-and anti-inflammatory findings and there have been similarities and differences in cytokine and immune cell profiles found between the brain, cerebrospinal fluid (CSF) and blood. In addition, the mechanisms involving the role of BBB-resident dendritic cells (DCs) in directing peripheral immune access across the endothelial and basement membrane barrier on the peripheral blood side, through the glia limitans on the brain parenchyma border, to the central parenchyma side of the BBB are still unclear (13, 14). This process appears to involve perivascular macrophages that accompany DCs at the BBB (13, 15). Regulation of the brain entry of T-cells by DCs and retainment of these cells in the parenchyma is subject to modulation by the chemokine and cytokine composition in the surrounding milieu. One of the main factors involved in this is an inhibitory effect driven by the cytokine transforming growth factor-beta (TGF-β) at the BBB (16). The priming of CD8+ T-cells to enter the brain occurs in the periphery (17) and attraction into this space is regulated by chemokines and antigen-responsive T resident memory (Trm) cells (18), driven by ligation of its CD103 receptor to complementary E-cadherin in the brain parenchyma (19).

Another factor which may be involved in the attraction of T-cells to the brain parenchyma is infection with viruses such as herpes simplex virus type I (HSV-1). This virus is known to have different effects in the brain and peripheral sites of primary infection on T-cells (20). In addition, secondary HSV-1 infection of DCs and perivascular macrophages may disrupt antigen presentation in the perivascular space and neurogenic zones of schizophrenia patients (20). This can result in long lasting consequences of a single hit of virus infection.

Here, we review the role of inflammation and viral infection as disease modifiers in schizophrenia. It is hoped that further studies in this area will lead to the identification of new biomarkers and drug targets to help move the field forwards and enable personalized medicine approaches to improve the lives of people suffering from this major psychiatric disorder.

## 2. Pro-inflammatory and anti-inflammatory findings in schizophrenia

**Figure 1:**
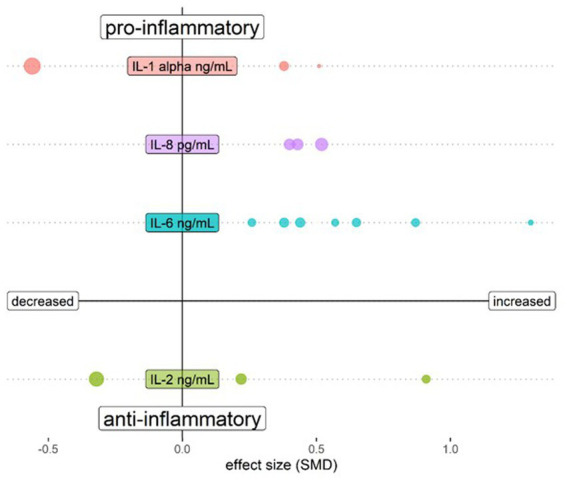
Bubble plot showing changes of cytokines assessed in CSF of schizophrenia patients compared to healthy controls. Both upper quadrants show the *pro*-inflammatory cytokines IL-1 β, IL-6 and IL-8 and the lower quadrants show *anti*-inflammatory cytokine IL-2. The left quadrants indicate a lower cytokine level in schizophrenia and the right quadrants a higher level. Each bubble represents a study and the magnitude of the bubble is calculated from the percentage of patients with the respective change of the cytokine relative to total patients assessed for the specific cytokine. Data are shown for cytokines tested in at least 3 studies in Orlovska-Waast et al., (26). Figure created with R-statistics 4.2.2.

The biological mechanisms of psychosis are reflected in the blood, cerebrospinal fluid (CSF) and brain parenchyma. In each matrix, increased amounts of multiple pro-inflammatory mediators have been discovered, albeit with variability possibly due to heterogeneous patient groups. This includes increased levels of interleukin (IL)-6, tumour necrosis factor (TNF)-α, IL-1β, and IL-12 (21) in serum of medicated patients, and increased concentrations of interferon (IFN)-γ, IL-6 and IL-12 in serum of unmedicated first episode patients [(22); Table 1A]. Most of the changes in blood were not found to be reflected in CSF in a meta-analysis addressing cytokine changes in both compartments, which only demonstrated a reduction of CSF IL-1β (23). In an analysis of a larger CSF dataset, both IL-6 and IL-8 were found to be significantly elevated in schizophrenia patients as compared to controls, an effect not found in affective disorder patients (26). Table 1B shows the individual CSF studies conducted to our knowledge thus far regarding CSF cytokines, with pro-inflammatory IL-6 standing out as increased in schizophrenia. Figure 1 shows a relative representation of the individual studies regarding CSF cytokine changes (26) in a bubble plot, indicating that anti-inflammatory IL-2 may also be elevated in CSF in schizophrenia. However, cytokine levels in CSF are lower than in blood and a recent study by Singh et al. demonstrated potential limitations in sensitivity of multiplex cytokine assays in CSF studies of mental disorders (27). This suggests that some published studies in this area should be re-evaluated.

**Table 1:** Inflammatory and viral biomarkers that may modify disease outcome in schizophrenia.

Study (ref)	Author	Patients vs. controls	SZ group	Main finding (direction effect)
A: Meta-analyses showing pro-and anti-inflammatory cytokine changes in large cohorts of SZ patients and controls				
(21)	Momtazmanesh et al., 2019	n/a n/a	Not further specified	**Elevated** IL-6, TNF-α, IL-1β, IL-12, TGF-β
(22)	Dunleavy et al., 2022	651 521	FEP + Unmedicated	**Elevated** IFN-γ, IL-6, IL-12, IL-17
(23)	Miller et al., 2011	117 275 (IL-6)	FEP + Unmedicated	**Elevated** IFN-γ, IL-1β, sIL2R, IL-6, IL-12, TNF-α,TGF-β
		156 373 (IL-6)	Acute recurrence	**Elevated** IFN-γ, IL-1β, IL1RA IL-6, TNF-α, TGF-β **Reduced** IL-10
(24)	Mazza et al., 2022	705 632	Not further specified	**Elevated** monocyte count
(25)	Halstead et al., 2023	13,952 10,969	Chronic or Acute	**Elevated in both groups** IL-1β, IL-1RA, sIL-2R, IL-6, IL-8, IL-10, and TNF-α

Study (ref)	Author	Patients vs. controls	SZ group	Main finding (direction effect)
B: Studies showing inflammation related changes in CSF and concurrent changes in peripheral blood of SZ patients				
(139)	Jeppesen et al., 2022	104 104	Not further specified	**Elevated** CSF/Serum ratio of IgG and Albumin **Elevated** CSF WBC count
(140)	Coughlin et al., 2016	11 12	Recent onset(x) + medicated	**Elevated** CSF IL-6 **Elevated** Serum IL-6
(141)	Schwieler et al., 2015	23 27	Medicated with olanzapine	**Elevated** CSF IL-6
(142)	Hayes et al., 2014	46 35	Unmedicated	**Elevated** CSF IL-8, CCL8
(143)	Sasayama et al., 2013	32 35	Chronic, Medicated	**Elevated** CSF IL-6 and Serum CSF, positive correlation Serum and CSF
(144)	Söderlund et al., 2011	26 30	Male FEP, medicated	**Elevated** CSF IL-1β, IL-6 and IL-8
(145)	Garver et al., 2003	31 14	Acute recurrence + unmedicated	**Not elevated** CSF IL-6
(146)	Van Kammen et al., 1999	61 25	Chronic + temporarily unmedicated	**Not elevated** CSF IL-6 **Elevated** Serum IL-6
(29)	Nikkilä et al., 2001	35 47	Acute + unmedicated	**Elevated presence** in CSF of activated macrophages and activated lymphocytes (cytology).
(27)	Singh et al., 2023	20 21	FEP, medicated	**No change** CSF IL-8 and MCP-1**Elevated** Serum MCP-1Study demonstrates limitations in the sensitivity of multiplex cytokine assays for CSF studies in mental disorders.

Study (ref)	Author	Technique	Location	Main finding (*proposed mechanism*)
C: Studies illustrating a sequence of events how immune cells may become retained in the SZ brain: Initiation with enhanced expression of endothelial attachment receptors and release of chemokines from the brain parenchyma that attract immune cells which express complementary chemokine receptors and finally attachment of immune cells in the brain parenchyma by binding to complementary checkpoint receptors.				
(10)	Cai et al., 2020	Immunohistochemistry	PFC	Increased endothelial expression ICAM1: *Attachment receptor is ready* Increased presence CD14+, CD16+ monocytes and CD163+ (PVM): *Attaching PVMs are present, migrate(d) into BBB*
(8)	Weissleder et al., 2021	Immunohistochemistry	Anterior caudate B	Increased cells expressing CD64+, CD163+: *Attaching PVMs are present, migrate(d) into BBB* Reduced expression of NOTCH and Wnt signaling pathways: *Inflammation associated reduction of neurogenesis in SZ*
(9)	Purves-Tyson et al., 2020	Q-PCR, western blot, immunohistochemistry	Midbrain	Increased ICAM1 expression: *Attachment receptor is ready to grab immune cells* Increased CD163+ cells around blood vessels and in brain parenchyma:): *Attaching PVMs are present, in BBB and migrated into parenchyma*
(44)	Zhu et al., 2022	Sequencing	(DL)PFC	Increased expression of CD163+: *PVMs migrate(d) into parenchyma* Increased CCL2 transcripts: *Attracts CCR2+ monocytes and T-cells for effective migration into brain parenchyma* Increased expression of CD86+: *Retains CTLA-4+ T-cells in the brain parenchyma* Reduced IL-8 transcripts: *Ambiguous role, because elevated in the high inflammation group*.
(37)	Bergon et al., 2015	Sequence data	Various brain regions/	Lower CX3CR1 transcripts blood and brain: *Ambiguous: CX3CR1 most expressed in microglia, reduced activation in SZ?*
(45)	van Kesteren et al., 2017	Immunohistochemistry	Various brain regions	Higher density of microglia: *Ambiguous: might be due to inhibitory phenotype*
(48)	Pietersen et al., 2014	Sequencing	STG	Increased TGF cascade expression: *Immunomodulation of entering immune cells*
(30)	Schlaaff et al., 2020	Immunohistochemistry	Anterior Hippocampus	More T-cell + B-cell clusters: *Mechanistically retained lymphocytes in the brain parenchyma*

Study (ref)	Author	Experiment	Main effect	Hypothetical role in SZ
D: Studies used in the main text for the stepwise development of the hypothesis that retainment of T-cells and PVMs in the brain of a subgroup of SZ patients can be explained by presence of HSV-1: TGF modifies immune response to a tolerant mode, which is dependent on Tregs, and triggered by (latent copies of) HSV-1, which may reside in antigen presenting cells (DC/PVM), and result in attraction and retainment of Trm (CD103+) in the brain parenchyma, but at the cost of cognitive decline and psychosis risks in HSV-1 seropositive patients, especially under social stress, which temporarily reduces (brain) cellular immunity.				
(53)	Graham et al., 2014	Viral brain infection, depletion Treg (West Nile Virus)	Retainment of (Trm) CD103+ is dependent on Treg and TGF production	Retainment of T-cells in certain inflammation cases might be dependent on TGF.
(60)	Diaz et al., 2006	Human lymphocyte proliferation response *in vitro* to viral antigen	Treg depletion increases response against HSV-2	Immune tolerance for HSV might dependent on Tregs and explain certain inflammation cases
(61)	Sehrawat et al., 2008	Conversion of splenocytes into Tregs by TGF in HSV-1 infection	CD11c (DC) produced TGF favours differentiation into Treg.	DCs in brain might produce TGF modify T-cell differentiation *in situ* for virus tolerance
(63)	Prasad et al., 2019	Viral brain infection models	TGF is highly expressed in viral infected brain and induces (Trm)CD103+	TGF might be tolerizing latent viral brain infections in some patients with SZ.
(20)	Mikloska et al., 2001	Differentiate human monocytes into DCs while infected with HSV-1	HSV-1 reduces the T-cell stimulatory function of DC	HSV-1 might be present in DCs in (a subgroup) of inflammatory cases in SZ and modify T-cell retainment in brain
(68)	de Jong et al., 2008	Expose human (*in vitro* differentiated) DCs to HSV-1	DCs bind and internalize HSV-1 after binding to DC-SIGN (CD209)	Infected DCs might circulate, might be present at the BBB in specific patients with SZ
(68)	Jin et al., 2011	Immature human DCs were transduced with HSV-1 viral ICP34.5 gene	Transduced DCs express less co-stimulatory molecules after stimulation	Viral product ICP34.5 might be important in virus tolerance of DCs in blood and brain and modify SZ outcome
(71)	Smolders et al., 2013	Post-mortem human brain staining on T-cells (CD8+)	T cells inhabitate the perivascular space and parenchyma	T-cells may transmigrate into the BBB and parenchyma as potential sentinels for infection response
(72)	Khanna et al., 2003	Immunodominant epitope expressing Trm (CD69+) adoptic transfer to HSV-1 infected neuronal ganglion	Trms prevent reactivation of HSV-1. Immunodominant T-cells creating an immunological synapse with neurons	This study suggests that Trm might suppress HSV-1 neuronal infection effectively, if present in brain
(58)	Smolders et al., 2018	Post-mortem human brain staining on T cell subsets, notably CD103+ and co-stimulatory molecules. No psychiatric, no infection subset.	CD103+ (Trm) are associated with a reduced expression of differentiation markers, increased expression of tissue-homing chemokine receptors and low expression of cytokines	This study suggests an important migratory capacity of Trms in the healthy brain, as sentinels for local response to infection.
(92)	Mendez-Samperio et al., 2000	Infection of differentiating human monocytes with HSV-1	Human monocytes support infection and produce and shed TGF-β	TGF might be produced by monocytic lineage PVM, DCs at the BBB as response to HSV-1
(96)	Pollara et al., 2003	Infection of differentiating human monocytes with HSV-1	Infected monocytes are suppressed in antigen presentation and T-cell activation, but in seropositive individuals, LPS induces high IL-12 secretion. Lowest viral infection (MOI 0.3) gave maximum effect.	Serostatus of HSV-1 may modify the T-cell response at the BBB when DCs are infected with HSV and explain different immune responses in SZ patients. Lower MOI of DCs, might result in higher response with IL-12
(97)	Song et al., 1999	Infection of rat brain with HSV-1, via peripheral (cornea) versus central (brain injection) route	Immunohistochemistry positive for HSV-1 in hippocampus when peripheral route used.	HSV-1 latency might relate to hippocampal dysfunctions regarding neurogenesis in SZ
(98)	Menendez et al., 2016 xx	Infection of mouse brain with HSV-1 *via* peripheral route	After resolution of infection, replication genes of HSV-1 remain selectively expressed in neuronal progenitor cells in ependyma of hippocampus	Replication genes of HSV-1 might be expressed in certain inflammatory SZ cases. Capsid gene VP5 might be a promising candidate
(103)	Dong-Newsom et al., 2010	Social disruption stress in the light of HSV-1 latency after peripheral infection in mice	Increases expression of viral protein ICP0 peripheral, decreases ICP0 expression in neuronal ganglion (migration of DCs to ganglion)	Response to social stress in SZ patients may be modified by DC trafficking dependent on HSV-1 serostatus of patients.
(104)	Freeman et al., 2007	Social stress in HSV-1 infected mice, response of CD8+ T-cells	CD8+ T Cells become depleted after stress and cause activation of HSV genes.	Response to social stress in SZ patients may be modified via depletion of CD8+ cells and dependent on HSV-1 serostatus.

(A) SZ, Schizophrenia; FEP, first episode psychosis. Pro-inflammatory cytokines are indicated in black text, anti-inflammatory cytokines in red. (B) SZ, Schizophrenia; FEP, first episode psychosis; CSF, cerebrospinal fluid, (x) Recent onset: within 5 years of diagnosis. All studies are case-controls and most focus on cytokine IL-6. (C) PFC, prefrontal cortex; STG, Superior Temporal Gyrus; (DL) PFC, (dorsolateral) prefrontal cortex; PVM: perivascular macrophage. (D) DC, Dendritic Cell; Trm, Resident Memory T-cell.

Activated T-cells were significantly more abundant in schizophrenia patients in blood, with higher percentages of CD3 + CD25+ T cells and pro-inflammatory Th17 cells (28). In a study by Nikkilä et al., lymphocytes and macrophages were found to be aberrantly activated in CSF (29). Even in the brain parenchyma, signs of an ongoing immune response occur. As examples, an overexpression of endothelial cell intercellular adhesion molecule 1 (ICAM1) has been observed in frontal cortex of a subgroup of schizophrenia patients with signs of inflammation (10), and significantly more CD163 expressing perivascular macrophages were identified in subependymal tissue and in the midbrain of a different schizophrenia patient group (8). Moreover, occurrence of T-cell and B-cell infiltrates was found to be more prominent in a subgroup of schizophrenia patients, compared to affective disorder and non-psychiatric controls (30).

The first neuroinflammation imaging studies with positron emission tomography (PET) in psychiatry suggested increased activation of microglia (resident macrophage-like cells in the brain parenchyma) in patients experiencing a psychotic episode (31), or after a first psychotic episode (32). Both studies measured microglia activation with the translocator protein (TSPO) ligand [^11^C]PK11195, using the robust arterial blood sampling method as reference. Later studies, with [^11^C]PK11195 without burdensome arterial sampling proved less reliable (33). Current studies with more sensitive tracers such as [^11^C]PBR28 showed the opposite, with reduction of microglia activation in schizophrenia patients (34). However, use of different TSPO ligands might have different outcomes, because TSPO binding differentially involves microglia, astrocytes, endothelium and even neurons (35). Although the debate about reduction or increase of microglia activation seems to have been won at present by Plaven-Sigray with decreased binding of modern TSPO ligands in schizophrenia (36), this author states “evidence against increased TSPO from PET studies should not be taken as evidence against a pro-inflammatory immune state in schizophrenia, and we therefore agree with Marques et al., that the discussion of increased microglia activity should be kept open.” Without choosing a side in the discussion, TSPO activation is not inconsistent with the hypothesis that schizophrenia is associated with a pro-and anti-inflammatory state. The latter is associated with enhanced transforming growth factor beta (TGFβ) production by astrocytes which is related to impaired functioning and control of Treg cells that leads to IL-6 increase in the CSF as postulated by Corsi-Zuelli et al. (6). The Corsi-Zuelli and Deakin hypothesis will be discussed further in Section 3.

Interestingly, there are also clear signs of hypo-inflammation in the blood, CSF and brain compartments in schizophrenia patients (Tables 1A–C). For example, the anti-inflammatory transforming growth factor (TGF)-ß has been shown to be increased in serum of patients who typically have elevated serum pro-inflammatory cytokines, as described above (21–23). In the above-mentioned meta-analysis of CSF, no inhibitory cytokines, including TGF-ß, were found to be altered (26). Again, such findings should be interpreted with caution as many of these molecules may be present in CSF at unmeasurable levels using existing multiplex immunoassay platforms (27). According to a study by Drexhage et al., T-cell activation has both a pro-inflammatory and an anti-inflammatory signature due to the observation of increased anti-inflammatory CD4 + CD25highFoxP3+ regulatory T cells and IL-4+ lymphocytes in the peripheral blood of schizophrenia patients (28). It is also interesting to note that CX3CR1, a chemokine receptor important in the recruitment of immune cells to sites of infection or auto-immunity, is decreased in schizophrenia (37) and this is expressed on the endothelial side of the blood brain barrier (BBB) (38, 39). Corsi-Zuelli and Deakin’s hypothesis (6) suggests that a hypofunctional state of Tregs is central for both chronic peripheral low-grade inflammation and glial dysfunction in schizophrenia. Treg failure may result in the overproduction of TGFβ from astrocytes, inducing microglia toward a synaptic pruning state without active inflammation.

An interesting genetic study described the role of gain of function mutations of the C4A/C4B genotype, which have been identified in patients with schizophrenia (40). Since complement C4 signalling is upstream of the TGF-ß activation cascade (41), a gain of function mutation of the complement C4 system could facilitate the anti-inflammatory effect of TGF-ß and, in this way, switch the priming of T-cells toward tolerance in the course of antigen presentation by macrophages (42). This raises the question of whether activation of the TGF-ß signalling cascade might be a protective response necessary to survive a trigger for psychoses. On the other hand, it might be case that activation of this pathway makes the population more vulnerable to the development of psychoses and ultimately to the diagnosis of schizophrenia. An interruption of the TGF-ß cascade can lead to maldevelopment of the brain since the brain morphogenetic SMAD proteins 3 and 4 are downstream signal transducers in this pathway (43).

Another factor which remains unresolved regarding pro-or anti-inflammatory status in schizophrenia is the finding that microglia resident in the brain parenchyma express less of the complement 4 receptor (CR4/CD11c) (44). This is in contrast to the increased perivascular macrophage expression of CD163 shown in the same study (44). Reduced CR4 receptor expression in microglia might indicate less downstream effects of TGF-ß signalling on these cells, and thus an impaired inhibition of a pro-inflammatory response in schizophrenia, despite higher TGF-ß action.

## 3. Comparison of inflammation-related findings between brain and peripheral blood

The different levels of action of biological factors in brain, CSF and blood of schizophrenia patients are still clouding a clear view of the etiopathogenesis of this mental disorder. At the heart of understanding schizophrenia is what happens in the brain whereas the changes occurring in the CSF and blood appear to reflect events in the periphery. However, the changes in the brain are ambiguous with regards to promoting or fighting inflammation. Imaging and post-mortem analyses of brains from schizophrenia patients have revealed that: (a) endothelial cells expressed less of the chemokine receptor CX3CR1 (37) and more ICAM1 (10); (b) perivascular macrophages expressed more CD163 (44); and (c) microglia expressed less CD11c (44). However, microglia (45) and CD-3 T-cells (30) were found to be more prevalent in schizophrenia post-mortem brains than in unaffected controls (Table 1C).

The changes in the blood and CSF are similar in ambiguity. Pro-and anti-inflammatory actions act in concert at the T-cell level with increased inhibitory Treg and inflammatory Th17 cells (28), which may signal an unresolved brain infection that needs both of these arms of the immune system, or an ongoing auto-immune process (46). High TGF-ß in brain is associated with lower cortical thickness and cognitive decline, possibly due to regulation of an inflammatory or infectious trigger within the brain (47). TGF-ß and immediate downstream targets like bone morphogenetic protein (BMP) have been shown to be significantly upregulated in schizophrenia (48). This upregulation of TGF-ß might be directly responsible for a downregulation of microglia receptors such as CD11c by making them quiescent (49) without effect on the number of microglia (45). This observation is in line with the hypofunctional Treg hypothesis of Corsi-Zuelli et al. (6), which suggests that failure of Treg-cells may result in overproduction of TGF from astrocytes. This would lead to downregulation of (CD11c) expression of microglia, and create a milieu with more synaptic pruning, without encouragement of inflammation.

A peculiarity of brain immunity is that antigens from the brain are taken up by DCs in the parenchyma (50). These antigen-loaded dendritic cells are transported to peripheral immune organs such as cervical lymph nodes and spleen, where they present the antigen to immature CD8+ T-cells cells that transmigrate from lymph node, blood and through the endothelium into brain parenchyma (50). The chance to selectively find the CD8+ T-cell trafficking to the brain is negligible in the face of many immune processes going on, and trafficking is only reliably detectable in appropriate animal models (50). However, the cells central to the entry of CD8+ cells into the brain (DCs, perivascular macrophages) can be investigated easily by post-mortem research, and may show changes reflecting antigen presentation of specific epitopes matching environmental factors regulated by multiple arms of the cellular and humoral immune system, both requiring tolerance and suppression. Tolerance to foreign antigens can be triggered by the action of both TGF-ß (51) and IL-10 (52), with TGF-ß possibly originating from peripheral monocytes transmigrating into the perivascular compartment (16). Treg-dependent production of TGF-β contributes to the retention of CD8+ T-cells in the brain and acquisition of a resident memory phenotype (CD103, CD69) after exposure to TGF-β, which keeps them in the brain parenchyma as sentinels for long-lasting adaptive immune responses (53).

## 4. Role of cells that guide access of the peripheral immune system to the brain

CD11c antigen-presenting DCs can bypass the BBB and by traverse the basal lamina of the vasculature (13), express CX3CR1 on the endothelial lumen (39), and cross the glia limitans on parenchymal side. CD11c DCs are necessary and sufficient for immune presentation of foreign epitopes to T-cells, and for transmigration of these primed T-cells into the brain parenchyma (17, 54). In some cases, this priming can be detrimental and lead to autoimmune neuroinflammation.

**Figure 2:**
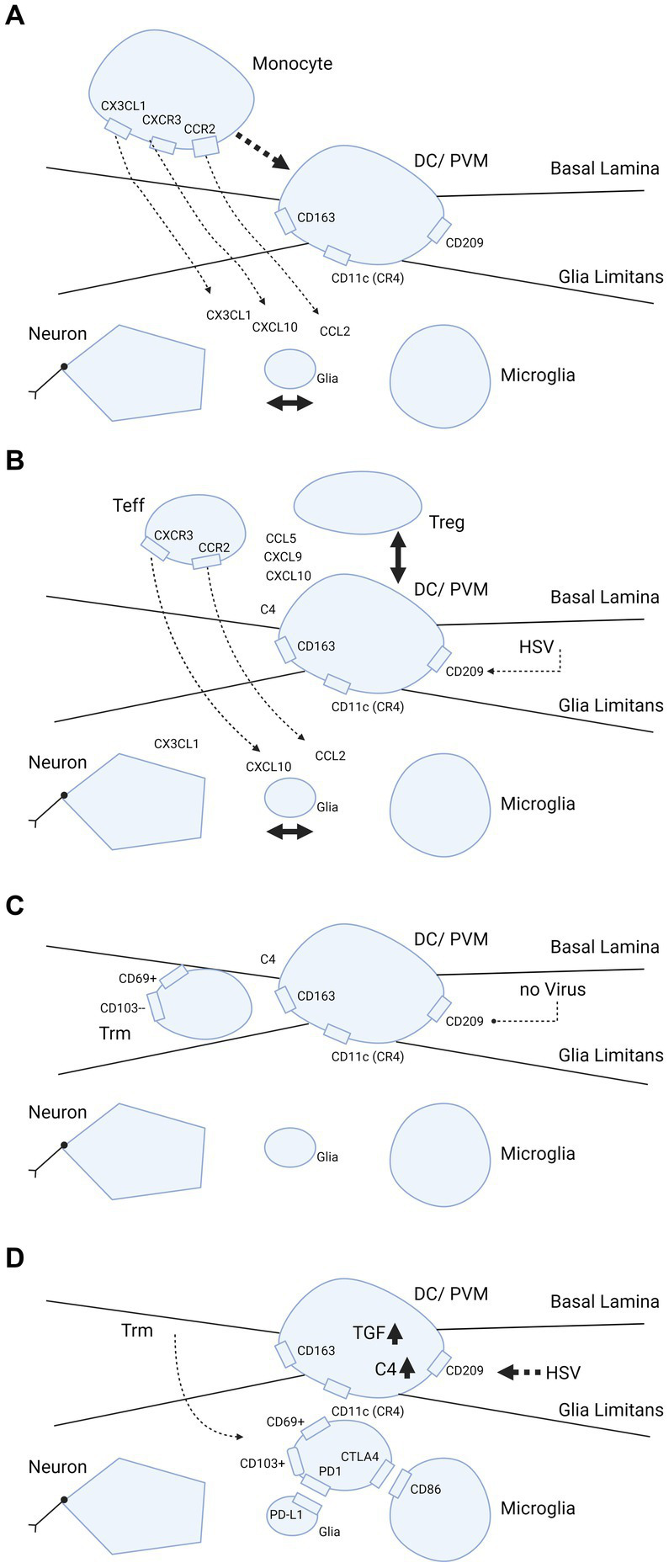
Trafficking of myeloid cells and T-cells into brain parenchyma and hypothetical dependence retainment of T-cells on immune modulation by HSV. **(A–D)** Cascade of events illustrating what happens when brain parenchyma becomes involved in intracellular viral infection. The consecutive steps are: **(A)** release of chemokines and attraction of monocytes from the blood by interaction between neurons, glia and microglia; **(B,C)** strategic positioning of monocytes differentiated into DC/PVM in the BBB; and **(D)** eventual transmigration of T-cells into brain parenchyma dependent on HSV. In further detail, **(A)** primary response after parenchymal infection, production of CCL2, CXCL1, CXCL10 chemokines that attract monocytes from the blood compartment with binding to their counter-receptors (CCR2, CXCR1 and CXCL10, respectively) on the monocyte cell surface. **(B)** Positioning of the monocyte as differentiated DC/PVM into space between basal lamina (endothelial basement membrane) and glia limitans (parenchymal basement membrane). With positioning within the BBB, this cell expresses CD11c (CR4, complement receptor 4), CD163 and CD209. Again, chemokines released from brain parenchyma play a part in the positioning. **(C)** Retainment of T-cells in the BBB space. Without further parenchymal triggers and inflammatory processes going on in the brain parenchyma, these cells develop into Trm (responsive, high expressing grB) with a CD69+ CD103- phenotype (58). **(D)** Shows what may happen if HSV infects the DC/PVM and changes the milieu within the BBB. In this scenario, HSV-1 promotes production of tolerizing complement C4 and TGF-β, which may lead to transmigration of T-cells within the BBB into the brain parenchyma and differentiation into (unresponsive, low grB expressing) Trm-cells with a CD69+ CD103+ phenotype. This phenotype is preferentially retained in the parenchyma by binding to complementary receptors of glia and microglia. Trm-cells express PD1 and bind to PD-L1 on glia and Trm express CTLA4 that binds to CD86 on microglia. Figure created with biorender.com.

Human CD11c cells can be distinguished by the presence of CD209, which is the dendritic cell specific ICAM3 grabbing non-integrin (DC-SIGN) receptor (13, 17). CD11c/C209 dendritic cells are of myeloid origin and are probably replenished from the bone marrow. This is in contrast to microglia, which reside behind the glia limitans in the parenchyma and are stationary throughout the life span. In humans, the perivascular space is also inhabited by phenotypic macrophages that express CD163 (55), which corresponds to the monoclonal antibody ED2 antigen in rodents (56). It is not clear whether CD11c and CD163 share common ancestors from a bone marrow CD34+ progenitor and/or if they are interchangeable when differentiating into each other. Perivascular macrophages are present in the perivascular space in human brain in the non-inflammatory state and exclusively express CD163 under these conditions (55). Perivascular macrophages are replenished from the bone marrow (57). In multiple sclerosis patients, microglia may also bear CD163 when they become activated [(55); Figure 2A].

Both CD11c DCs and CD163 perivascular macrophages connect the peripheral and central nervous system (CNS) immune systems. This process serves to regulate transmigration of primed T-cells into the brain parenchyma, and determines whether local antigen presentation leads to a vigorous antigen response or tolerance (59). It is also dependent on the extracellular TGF-ß and IL-10 composition as this regulates T-cell differentiation into activated CD69, CD103 Trm cells (18). In situations where there is no strong anti-inflammatory protection such as those caused by HSV-1, an effective and tissue sparing immune response against severe infections is possible (53). This also is true for corneal (60) and skin infections (61). Thus, the main role of perivascular antigen-presenting cells is to balance the pro-and anti-inflammatory responses. In viral infections, the balance between priming and tolerance is important. During virus replication, the former is predominant, but the latter takes precedence during the latency stage. In models of severe (auto)immunity, pro-inflammatory invasion of T-cells predominates (54). Similarly, in severe infections of the CNS, pro-inflammatory mechanisms predominate (62).

In schizophrenia, mRNA transcripts involved in the activation and presence of CD163 perivascular macrophages in the parenchyma have been shown to be significantly elevated in ependyma (8), midbrain (9) and frontal cortex [(10); Table 1C]. In addition, complement (C)1qA, C3 and C4 transcripts were also found to be increased. C4 deficiency is involved in intraparenchymal infections and in autoimmune conditions such as systemic lupus erythematosus (SLE) (63). Furthermore, DCs produce all complement factors, which creates an anti-inflammatory tolerant milieu via Treg-cell differentiation and increased production of TGF-ß and IL-10 (64). C4 probably acts upstream of the cascade leading to TGF-ß production (41). Thus, it could be that a potential tolerance-acquiring role of C4 exists in the midbrain in schizophrenia for accommodation of latent viruses like HSV-1.

## 5. Regulation of brain entry of T cells by DCs

CD11c DC transgenes expressing the green fluorescent protein (GFP) under the CD11c promoter have been used to characterize DC trafficking in mouse models (15, 17, 18). These models show that antigen response in the brain parenchyma is a three step process. First, the antigen is taken up in the brain parenchyma by the DC and transported to the periphery, cervical lymph node and spleen. In these lymphoid organs, DCs present antigens to CD8+ T-cells, which are primed and then return via the blood stream to the site of antigen presence in the brain parenchyma and express the CD103 receptor, qualifying the CD8+ cells as the Trm type. This sequence of events has been studied by experimental follow up of DC and T-cell migration after loading ovalbumin in DCs and injecting these into brains of mice (18). Multiple chemokines are produced by DCs as an attractant for T-cells. As examples, CXCL10 attracts T-cells via the CXCR3 receptor and CCL2 attracts using the CCR2 receptor, as demonstrated in animal models for multiple sclerosis [(54); Figures 2A,B]. The attraction of T-cells into the brain may not only rely on parenchymal production of CXCL10 and CCL2, but also on TGF-ß at the BBB. For transmigration of T-cells into the brain parenchyma and differentiation into Trm-cells, the production of TGF-ß by local DCs is crucial. Under the influence of TGF, CD69+ Trm-cells differentiate further *in situ* and show CD103+ expression, possibly bound to microglia-expressed E-cadherin and thus remain in the parenchyma behind the BBB (63). Along with TGF-ß, additional cytokines aid in the developmental program of Trm-cells (65). Failure of complementary binding between the CCL2 and CXCL10 chemokines and the associated chemokine receptors is associated with failure of immune control in HSV-1 brain infections (38, 66). CD11c DCs and T-cells that mature, are also bound by checkpoint receptors. After local priming or tolerance formation, the T-cells remain in the parenchyma and are retained there by the binding of checkpoint inhibitors programmed cell death protein 1 (PD-1) and PD ligand (L)-1, which are expressed in T-cells and glia, respectively (67).

Interestingly, DC-SIGN is an entry receptor for human immunodeficiency virus (HIV) and HSV-1 entry (68), and invasion by these viruses reduce maturation of DCs (20, 68, 69), which may impact the efficacy of antigen recognition, priming and tolerance at the interface of DC and T-cell communication, and trafficking of T-cells into the brain parenchyma. To our knowledge, this possibility has not yet been considered in scientific research.

## 6. Chemokine production and ligation to cells in the brain parenchyma determine retainment of T-cells

The corpus callosum has been investigated for the presence of resident CD8+ Trm-cells and for determining which surface receptors these cells express (70, 71). These cells can be subdivided into CD69+ CD103- and CD69+ CD103+ types. CD103+ is assumed to be the more differentiated type and its presence may be due to a previous viral encounter. It has been suggested that T-cell responses against viruses such as HSV-1 play a crucial role in this control (71). CD4+ and CD8+ T cells appear to congregate near HSV-1-infected neurons (72) and the release of IFNγ and granzyme B by CD8+ T cells leads to inhibition of virus reactivation (58). CD103+ cells are enriched for expression of the tissue homing chemokine receptors CX3CR1, CXCR3, CCR5 and CXCR6. CX3CR1 is essential for maturation of CD69+ CD103- cells into CD69+ CD103+ cells, which carry lower levels of the cytolytic granzymes B and K, and increased levels of the inhibitory receptors cytotoxic T-lymphocyte–associated antigen 4 (CTLA-4) and PD-1 [Figures 2C,D; (73)]. In inflamed brain scenarios, the counter receptors of CTLA-4 (CD86) and PD-1 (PD-L1) were found to be expressed in local microglia and glia, respectively (63 74). Both CD86 and PD-L1 have an inhibitory action on antigen priming and response to antigens, creating tolerance (73).

For full differentiation of Trm to CD8+ CD69+ CD103+ T cells, a three party interaction at the interface of the BBB is necessary. Immature CD8 + CD69 + CD103– cells need contact with the antigen presenting CD11c DCs (or CD163 perivascular macrophages) and CD4 + CD25 + FoxP3 Treg-cells. Importantly, Treg cells produce TGF-ß for tolerance (52, 74, 75) and CD11DC/CD163 perivascular macrophages (PVMs) interact with Trm-cells to provide the antigen which these cells are eventually primed against (Figures 2B,C). The Trm-cells retain some degree of tolerance needed for a prudential antigen response *in situ* by exposure to TGF-β produced by Treg cells. CD11c DCs are sufficient to prime T-cells for full development into the CD103+ cell type (15, 18). However, this can lead to destruction of brain parenchyma, most likely because the response is not simultaneously inhibited by presence of the third party Treg-cells.

In viral infections with the neurotropic virus HSV-1, antigen presentation at the BBB might also involve the three-way interaction described above, leading to tolerance via TGF-ß synthesis and primed CD8 + CD69 + CD103+ Trm-cells [Figure 2D; (63)]. Retention of tolerant Trm-cells in the brain occurs by the bilateral interaction of CD103+ with microglia-expressed E-cadherin. This is driven by the presence of IL-4 (63), PD-L1 and PD-1 (67). In peripheral infections such as those caused by HSV-1, the process is regulated by Trm-cells, which are activated when DNA replication occurs (76). In brain infections, other rules may apply (addressed in the next section).

A recent study identified increased presence of CD163 perivascular macrophages in the frontal cortex of schizophrenia patients (10). It is possible that this scenario provides an attractant for invasion and retainment of T-cells in the brain, consistent with the findings of a study by the Steiner group (30). In addition, the increased presence of human leukocyte antigen-DR (HLA-DR) antigen-presenting microglia found in an earlier study by the same group (77) may reflect the perivascular macrophage/DC population active in schizophrenia.

## 7. Parenchymal HSV-1 presence may lead to neuro-immune alterations in brain parenchyma

There has been conflicting evidence for a role of viral infections in psychiatric disorders. A very large post-mortem brain study by Min et al. found no increased persistance of CNS-related viruses in schizophrenia, bipolar disorder and autism spectrum conditions (78). In addition, the presence of viruses such as HSV-1 in the brains of healthy individuals (79, 80) poses a problem in understanding how this might occur without leading to severe inflammatory disease during their lifetime. In patients who have experienced a full blown herpes encephalitis, the viral DNA remains present (81) and primarily infects the temporal lobe and hippocampus (82). This potentially explains the long-time irreversible memory problems observed in some of these patients (82–84). In schizophrenia, HSV-1 seropositivity has been associated with cognitive decline or impairment (85). However, serostatus only reflects an experienced infection in the personal history and in the majority of cases the infection remains peripheral and latent in the trigeminal ganglion. In healthy individuals, seropositivity to HSV-1 has also been associated with reduction in cognitive functioning (86). However, the presence *per se* of HSV-1 antibodies in the periphery of even the CNS does not predict nor explain fully the occurrence of psychosis in vulnerable subjects. To demonstrate a role of HSV-1, other factors must be taken into account and the immunological changes in brain, CSF and blood that are operative in schizophrenia should be seen as consensual with a virus-driven explanatory framework.

Specific findings in a schizophrenia study support the case for viral involvement. A recent study showed that treatment of patients with psychosis with a high dose of valaciclovir (8 grams per day, for 1 week) reduced TSPO binding as an indication of microglia activation state in the hippocampus and other brain regions (87). As valacyclovir is incorporated into polymerizing viral DNA and not host DNA, the above findings suggest the presence of actively replicating viral DNA during psychosis in these individuals. The involvement of multiple brain regions and the vulnerability of neurons for an apoptotic response against a replicating virus suggest that infection occurs in other cell types such as glia, microglia, PVM-DCs or epithelial cells. Since TSPO binding occurs primarily in macrophage monocytic lineages, the most probable explanation is an effect of valaciclovir on virus replication within PVM/DCs. It should be noted that unlike neurons, glial cells can still proliferate and, therefore, might be responsible for the reduced TSPO-binding following acyclovir treatment (88).

### 7.1. The role of TGF-β in neuroinflammation

A study by Corsi-Zuelli et al. suggested that containment of cerebral inflammation by local production of TGF-ß induced by Treg-cells plays a central role in schizophrenia (6). This may explain the seemingly disparate findings regarding neuroinflammation and may lead to new avenues of research toward the etiopathogenesis of this psychiatric illness. For example, the presence of HSV-1 DNA in the nucleus may modify the immune response *in situ* in the brain as an actively transcribed episome in host DCs and perivascular macrophages. Given that HSV-1 can have profound effects on the perivascular DC phenotype, this will lead to flawed maturation of these cells (20, 68, 69) which, in turn, would have altered effects on neuronal function and survival [Table 1D; Figure 2D; (89)].

The natural production of TGF-ß could drive a counter-regulatory response against profound lytic infections otherwise marked by prevailing pro-inflammatory mechanisms, as in a fulminant herpes encephalitis (90). In these cases, the current clinical protocol calls for timely antiviral treatment such as high dose acyclovir. Despite this, mortality remains high at approximately 20% in the first days of referral (91). The finding that monocytic myeloid DC and PVM precursors that contain replicating HSV-1 produce TGF-ß (92), suggests that this may be a coping mechanism of the virus which allows it to remain in a tolerant milieu though induction of Treg-cell differentiation (93). However, this counter-regulatory response could have its costs, including a potential loss of cortical volume (47). Also the reduced expression of CX3CR1 in schizophrenia could be the result of exhaustion due to an imbalanced immune response.

### 7.2. Factors affecting the balance of pro-and anti-inflammatory responses in schizophrenia

The imbalance between the pro-and anti-inflammatory responses in schizophrenia may be viewed as a chronically stalled state due to the presence of a non-eradicable pathogen in brain parenchyma. The increased prevalence of schizophrenia in people with gain of function mutations in the complement C4A/C4B gene cluster (40) might provide an explanation for this. C4 is instrumental in expression of TGF-ß (41) which is hypothetically linked to creating a balance between pro-and anti-inflammatory mechanisms suppressing expression of a potentially lytic response against infected neurons. This process is facilitated with the help of C4-tolerant macrophages (94) and adequate virus responsive Trm-cells in the brain (95). The presence of HSV-1 in key positions of the immune system in the BBB might not only act to increase the expression of TGF-β (92), but also influence downstream events such as the SMAD3 and SMAD4 signalling, essential for neurodevelopment during embryogenesis and later stages of life (43).

Immune stalling of virus-infected DC and/or PVM cells (96) might explain the concurrent release of pro- (IL-6 and IL-8) and anti-inflammatory (IL-10 and TGF-ß) cytokines in schizophrenia. In addition, the infected cells might act in favor of a pro-and anti-inflammatory cellular response through activation of Th1/Th17 helper and Treg-cells, respectively (28). The central role of TGF-β in the balancing act between suppression against reactivation (58) and prevention of a lytic response will keep the infection at subthreshold levels (92). In non-replicating neurons, this stalled pro-and anti-inflammatory action can be advantageous since lytic infection and latency is forestalled. In replicating cells, the virus can be transmitted after division to both daughter cells by peripheral and central routes (97) and may remain indefinitely due to ineffective clearance by surrounding T-cells and the ambiguity of the immunological response (98). Neurogenesis occurs in ependymal cells (99) and this process is impaired in schizophrenia (11) in association with elevated PVM cell CD163 expression (8). Therefore, looking for presence of viral DNA in these cells may be a viable option for further research regarding the aetiopathogenesis of the aberrant pro-and anti-inflammatory immune alterations in schizophrenia patients. Studies thus far have found HSV-1 DNA in the ependymal niche and in perivascular sites where DCs and PVM cells or their transcripts accumulate (8–10, 44, 100).

### 7.3. HSV-1 infection and schizophrenia

**Figure 3:**
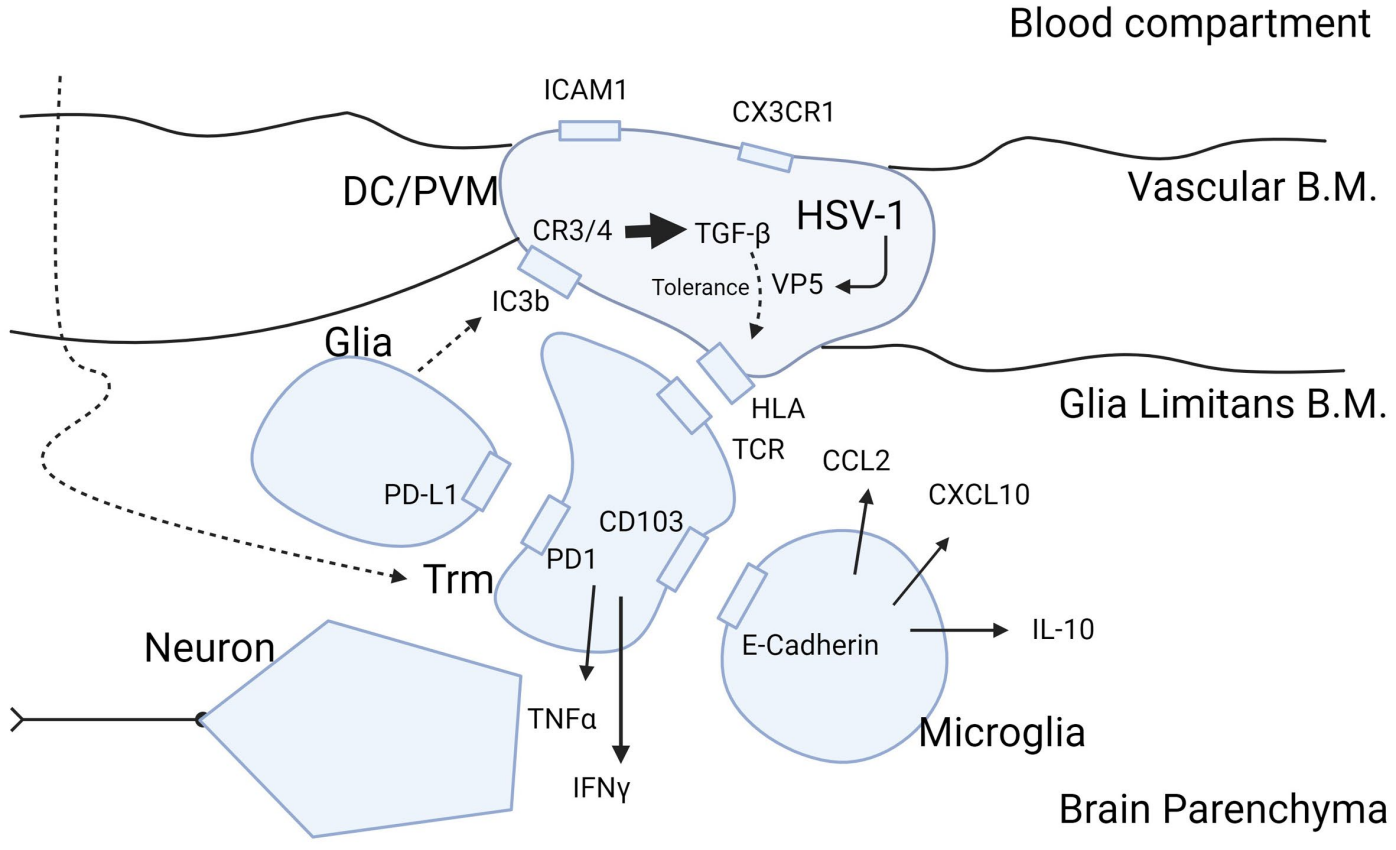
Molecular insight into HSV-1 protein expression as potential cause of inflammatory ambiguity at the BBB in Schizophrenia. Infection of the BBB spanning DCs and PVM cells with production of the immunogenic protein VP5 by HSV-1 may explain a stalled immune response in brains of patients with schizophrenia. The figure highlights the sequence of events in case of viral infection of brain parenchyma and attraction of T-cells into the brain after priming in peripheral immune organs such as submandibular lymph nodes and spleen. Entry of T-cells from blood into the brain parenchyma is mediated CCL2 and CXCL10 produced by microglia (110). T-cells mature during transmigration through the BBB into the parenchyma (50) in their trajectory (large curved dashed arrow) to Trm cells by exposure to inhibitory cytokine TGF-ß (63) produced by DC/PVM cells (16, 92), possibly triggered by HSV-1 (81). Trm cells are locked into place by the PD-1/PD-L1 and CD103/E-cadherin interaction with glia and microglia, respectively (63, 67). We hypothesize that retainment of T-cells in the parenchyma in schizophrenia is perpetuated by the DC/PVM virus-infected phenotype. This leads to entry and retention of T-cells into parenchyma, and continuous presentation of antigen to intraparenchymal T-cells to secure the brain in a virus-tolerant mode. The mode of tolerance of DCs and PVM cells is acquired by complement C3 and C4 receptors, triggered by the cleavage product of C3b (iC3b) generated by glia (111). The action of iC3b on the DC/PVM generates tolerance for viral protein VP5 in the DC/PVM by intracellular production of TGF-ß (112). This modifies the human leukocyte antigen (HLA)/major histocompatibility complex (MHC) interaction of the DC/PVM with the T-cell receptor in a tolerance mode. This does not preclude expression of pro-inflammatory cytokines IFN-γ and TNF-α but balances their potentially detrimental effect on neurons. TGF-β may also modify the DC/PVM expression of ICAM1 and CX3CR1 at the endothelium as occurs in schizophrenia (10, 37). B.M., basement membrane; Trm, tissue resident memory; DC, dendritic cell; PVM, perivascular macrophage; HLA/MHC, human leukocyte antigen/major histocompatibility complex; HSV-1, herpes simplex virus 1; VP5, HSV-1 viral protein 5. Figure created with biorender.com.

Perpetual HSV-1 infection of the ependymal cells in schizophrenia would fit the neurodevelopmental and multiple hit hypothesis (101). This suggests that multiple hits of stress-related moments in life can lead to a psychotic episode. The first hit could be *in utero* by direct viral exposure of the fetus, or by the immune response of the mother to fight off viral infections. Both of these may modify brain development and Treg cell maturation. If Treg cells become hypofunctional after birth, this may make the individual more vulnerable for consecutive inflammatory of infectious hits, that need “healthy” Treg responses (102). Such stressors could reduce resistance as a potential mechanism that eventually triggers a disastrous cascade of events in brain tissues containing latent virus. In support of this, social disruption stress in rodents has been shown to increase the presence of antigen presenting cells toward latent HSV-1 (103), and restraint stress was found to impair CD8+ T-cell responses to latent HSV-1 (104). An important neurogenic zone in adults is the subventricular/subgranular zone of the hippocampus, which is vulnerable to inflammation (105). Neural stem, or B-cells, extending into the ependyma provide progenitors for neurons in these zones in the hippocampal dentate gyrus region (106). Therefore, infection of ependymal cells may have profound influence on maturation and differentiation into amplifying progenitor cells and neuroblasts. From the superficial ependyma toward the deep vasculature, CD209-expressing DC and PVM cells regulate the milieu involved in the differentiation processes (107). HSV-1 infection of antigen presenting cells may influence neurogenesis through modification of cytokine release or via shedding of virus protein-loaded exosomes. When antigen presenting cells are infected with HSV-1, the IFN-α response is mitigated and that of IFN-γ is elevated, dependent on presence of the infected cell protein (ICP)34.5 (70). ICP34.5 colocalizes with proliferating cell nuclear antigen (PCNA) in the nucleus to shift the DNA damage effect toward a viral replication response (108). In conjunction with gC1Qr/HABP/p32, ICP34.5 is essential for shedding of viral gene products to the extracellular space, and the complex formed between ICP34.5 and gC1QR/HABP/p32 aids in the nuclear egress of the ICP34.5 protein (109). The latter facilitates further egress of the viral proteins VP5/ICP5 via exosomal release [Figure 3; (113)].

### 7.4. Similarities with SARS-COV-2 infections

It is also likely that a similar mechanism plays a role in the manifestation of neurological and neuropsychiatric conditions such as depression, sleep disorders and schizophrenia following infection by other viruses such as SARS-CoV-2 (114–120). A recent transcriptome profiling study of post-mortem brains of SARS-CoV-2-infected patients by Yang et al. showed that peripheral T cells had infiltrated the parenchyma, and microglia/astrocyte subpopulations, and mimicked pathological states previously identified in human neurodegenerative diseases (121). Another study of a peripheral blood cell dataset from SARS-CoV-2-infected patients identified an overlap of immune-related genes from patients with bipolar disorder, schizophrenia and major depression (122). The overlapping genes including components of the TGF-β, (MAPK) and peroxisome proliferator-activated receptor (PPAR) signalling pathways, thus implicating perturbations in inflammation cascades. Although these studies suggest an overlap between the mechanism(s) of how viruses such as HSV-1 and SARS-CoV-2 can lead to neuropsychiatric complications, further research is required to gain further insights into this process.

## 8. Limitations

It should be noted that there are a number of assumptions in this hypothesis and applicability to all psychoses is not possible. Another limitation is that T-cells present in brain parenchyma (30) are not only present in patients with schizophrenia, they may also be present in depressed patients and healthy controls. In cases where T-cells are present, this does not automatically indicate presence of HSV and could also be indicative of Epstein–Barr virus, toxoplasma or SARS-CoV-2. Another potential caveat concerns the leaky VP5 protein of HSV, which may not be the only immunogenic protein involved in creating a chronic tolerizing interaction between DC/PVM and T-cells. This could also be auto-immune mediated in relation to an infection that has resolved but still leaves a continuously activated (tolerant) immune response, between DC/PVM and T-cells. In addition, the tolerizing response with grB present in low quantities, might be lessened and lead to destructive changes with greater severity than expected in schizophrenia. Another limitation concerns the point that antigen presentation occurring at the BBB might also be regulated by peripheral factors, such as those occurring in the lymphoid organs.

## 9. Conclusion and future prospects

Viral particles such as those from HSV-1 may have an influence when situated at the interface of the BBB in antigen presenting cells that normally guard the brain by providing choices of priming or tolerance for invading T-cells toward latently present pathogens [Figure 3; (10, 16, 37, 50, 63, 67, 92, 110–112)]. The action of valaciclovir to reduce activation of PVM cells, DCs or microglia in patients with psychosis might tell us, that replication of the virus is ongoing in these patients in multiple brain regions. If indeed HSV-1 is present in these cells, it might be possible to detect the virus in post-mortem samples. Disparate changes in pro-and anti-inflammatory processes, central versus peripheral inflammatory changes in schizophrenia might be dependent on whether or not or where viral infection of the DCs occur at the BBB interface. As this may appear in only a proportion of patients, it could explain the variability and disparities found across different studies. It is also important in this regard for researchers to publish negative findings to add further information to this debate (123).

The hypothesis that PVM cells and DCs may be infected with viruses like HSV-1 in schizophrenia can be investigated in a similar manner as addressed in post-mortem investigations of infected neurons (124). This suggests the potentially useful approach of investigating T-cell areas in schizophrenia patient brains (30) and performing immunohistochemistry in adjacent sections for presence of HSV-1 in PVM cells and DCs. If the virus is present at low copy numbers, such as one per cell, the chance to find it and to test or falsify the hypothesis will be remote with currently used techniques. Held et al. revealed that neurons in the trigeminal ganglia detected as positive for the latency-associated transcript (LAT) by quantitative reverse transcription-PCR were always positive for HSV-1 miRNAs and DNA, regardless of whether these were surrounded by T-cells (124). Therefore, if sensitivity of detection of viral proteins by immunohistochemistry is needed, the choice of target must be a protein that is abundantly expressed during abortive replication of the virus and preferably more abundant than LAT. Alternatively a technique more sensitive than *in situ* hybridization (ISH), could be used.

In studies of CSF inflammation, it will be critical to characterize performance of the multiplex immunoassay platforms applied to avoid misleading results. We recently showed that it is essential to accurately determine the limit of detection (LOD) of the individual cytokine assays in these platforms as many of these molecules are present at <10 pg/mL concentrations in CSF and cannot be distinguished from the signals produced by blank samples (27). For this reason, we suggest following the Clinical and Laboratory Standards Institute (CLSI) protocol to increase confidence in the results through accurate determinations of the LOD and limits of the blank (LOB) (125, 126). Future studies on this topic should also consider the use of more sensitive assays for detection of low abundance cytokines in CSF which have sensitivities two to three orders magnitude greater than most existing multiplex immunoassay platforms (127–130).

The most elegant opportunity is determination of proteins expressed early in the abortive process of reactivation and renewed replication. In animal models of HSV-1, it has been determined which proteins are expressed first and most abundantly during the reactivation process. Surprisingly, the VP5 protein that is needed late in the process to assemble the full virion, is expressed at an early stage (131), possibly because it is needed in abundance for this stage of the virus replication process (132). Antibodies for use in immunohistochemistry studies are available against VP5, so this might a fruitful option to proceed with.

Finally, it should be noted that further elucidation of the pathways involved in the development of psychosis following viral infections can lead to the identification of biomarkers and drug targets which could be applied in personalized medicine approaches for the best possible patient outcomes. For example, patients could be tested for the presence of antibodies against a panel of viruses such as HIV-1 and SARS-CoV-2 as well as against a multiplex cytokine array to pinpoint the inflammation status. The later should include assays for both pro-and anti-inflammatory cytokines and other biomarkers which have been associated with neuroinflammation (Table 1). This approach could help to stratify patients and identify those who are likely to respond to specific antipsychotics or add-on anti-inflammatory medications (133–137). In line with this, standardized methods should be established for assessing the risk–benefit ratio of adding available immune-modulating drugs to standard psychiatric therapeutics (138). It is hoped that such an approach would lead to more effective patient care in the field of mental health.

## Author contributions

HK drafted the manuscript including Table 1 and Figures 2, 3. JS contributed to each version of the manuscript and added corresponding literature in each stage. PG added all information regarding COVID, drafted the summary and abstract and edited the English language of the whole manuscript as a native speaker. HD created the bubble plot, Figure 1. All authors have read and agreed to the final version of this manuscript.
